# Genetic association of MBL-2 gene polymorphisms with Filarial chyluria

**DOI:** 10.6026/97320630015806

**Published:** 2019-12-10

**Authors:** Shriya Pant, Apul Goel, Pravin Kumar Gangwar, Jyotsna Agarwal, Arvind Kumar Singh, Satya Narayan Sankhwar, Prashant Gupta

**Affiliations:** 1 Department of Urology, King George's Medical University, Lucknow, UP,India; 2 Department of Microbiology, Dr. Ram Manohar Lohia Institute of Medical Sciences, India; 3 Department of Microbiology, King George's Medical University, Lucknow, UP, India

**Keywords:** Mannose-binding lectin, lymphatic filariasis, chyluria

## Abstract

Lymphatic filariasis has become a significant public health issue in North India. The association of polymorphisms in MBL2 gene with filarial chyluria (FC) is evaluated in the North Indian patients for the first time. Hence, a tertiary care hospital based case-control study was conducted in north India where FC is endemic. Therefore, 186 confirmed patients of FC as cases and 210 age-, sex- and residence-matched subjects as controls were enrolled for the study. Filarial etiology was confirmed using diethylcarbamazine (DEC)-provocation test, immune chromatographic test and IgG/IgM antibody test. MBL2 gene polymorphisms at codon 54 and -221 promoter region were genotyped by PCR followed by RFLP. Wild-type, heterozygous and homozygous mutant frequencies of MBL2 genotype at the codon 54 were 57.5%, 32.8% and 9.7% in the case group and 62.9%, 30.5% and 6.7%, in controls, respectively. The same at the -221 position were 51.1%, 44.1% and 4.8% in FC patients and 44.3%, 40.0% and 15.7% in controls, respectively. Thus, results no significant association between MBL2 polymorphism at codon 54 and FC. However, polymorphism at the -221 promoter region is linked with FC with a significant odd-ratio of 0.27 (confidence interval at 95% was 0.12-0.59; p<0.001). This preliminary finding is intriguing for further confirmation using a larger study with more patients.

## Background

Chyluria is an uncommon and delayed manifestation of lymphatic filariasis (LF) and is characterized by the presence of chyle in urine resulting from fistulous communications between the lymphatics and the urinary tract [1]. It is estimated that around 1,100 million people across the world are living in endemic regions for LF and exposed to risk of developing infection. Of the total global burden, India contributes about 40% and approximately 50% of the residents at risk of filarial infection [2]. The World Health Organization declared LF as a "neglected" tropical disease initiating "The Global Program for Elimination of Lymphatic Filariasis" and set the target for the year 2020 for elimination [3,4]. Up to 10% of patients with filariasis develop chyluria. It is predicted that approximately 90% cases globally and more than 99% filarial cases in our country are due to the infection of the parasite Wuchereria bancrofti [5]. The factors that predispose a person to develop LF have not been investigated. It is still unknown as to why one member of a family develops LF while another is spared. As the chances of mosquito bite remains the same in endemic areas, the answer to predilection for LF probably lies in genetic studies. Unfortunately, genetic studies on LF are rare.

Mannose-binding lectin (MBL) protein is first-line of defense which is encoded by MBL2 gene that has a key role in activation of complement system and opsonization of pathogenic virus, bacteria and parasites. MBL2 gene is located on chromosome 10q21.1. There are six single-nucleotide polymorphisms (SNPs) in MBL2 gene out of which three SNPs are located in structural region of exon 1 [codon 54 (Gly-Asp), codon 57 (Gly-Glu) and codon 52 (Arg-Cys)], while the rest of the SNPs are in the promoter region [-550 (G>C), -221 (G>C) and +4(C>T). The variants of MBL2 gene can influence serum MBL levels and also have an effect on transcription of protein. It has been evaluated that MBL2 gene is associated with various infectious diseases including LF [6]. The evidence linking the association between MBL2 gene polymorphisms with occurrence of LF is scanty. After extensive literature review only two studies were found that have looked for this association. In a cohort study, reported by Meyrowitsch et al. (2010) a significant association was found between MBL2 gene variants and predisposition to Wuchereria bancrofti infection [7]. Choi et al. (2001) reported a case-control study that looked into association between MBL2 gene polymorphism and susceptibility of filarial infection [8]. However, no study has reported the association between filarial chyluria (FC) patients and MBL2 polymorphisms in North Indian population. Of all manifestations of filariasis, urologists primarily treat chyluria. So in our study we chose FC patients as cases. We recently reported on the role of CHIT1 gene in the predisposition of filarial chyluria (FC) [5]. Carrying the same work forward, we analyzed the possible association of polymorphisms in MBL2 gene with FC.

## Methodology

### Study design and subjects:

This case-control study was conducted between March 2013 and April 2016 at a tertiary-care center in north India in a region where filariasis is endemic. Ethical clearance was obtained from the Ethical Committee of our institute (Reference code number: 5534/R.cell-13). 198 chyluria patients were screened for confirmation of filarial etiology; 186 cases gave positive results for FC. After taking written informed consent, data was documented on a predesigned proforma. Patients aged more than 18-years with FC, diagnostically confirmed by specific tests were enrolled. Subjects with non-chyluric whitish urine, non-parasitic chyluria, uncontrolled diabetes, pregnancy, renal failure and any malignancy were excluded from the study. Patients with non-chylurial reasons for whitish-cloudy urine (like severe pyuria, amorphous urates, phos¬phaturia) were excluded. Filarial etiology was confirmed by using Giemsa-stained thick and thin smear examination followed by Diethylcarbamazine (DEC)-provocation test, [9] immuno-chromatographic card test (ICT) (BinaxNOW® filariasis, Alere, North America, Orlando, USA) [10] and IgG/IgM-combo rapid antibody test (CTK Biotech Inc., CA, USA) [11]. Patients found positive by any of these tests were confirmed to have FC. For comparison, subjects without any sign or symptoms of LF, found negative by ICT and IgG/IgM antibody test both, were enrolled as controls after obtaining written consent. The controls were recruited by following the criteria of matched age, sex, and geographical region to the cases. Most controls were family members/attendants of patients. However, as recruiting consenting controls is difficult, we occasionally included other healthy volunteers. Parameters obtained consist of demographics of subjects (age, sex, and ethnicity), details of chyluria like duration, grading of severity of current episode, [12] number of previous episodes, chylous clot retention, and details of investigations like hemoglobin, urinary parameters like urinary triglycerides and cholesterol levels. After collecting the above details, 5-ml blood sample was collected from each participant to perform diagnostic tests and genotyping.

### Estimation of sample size:

The sample size was estimated on the basis of reported prevalence of genetic polymorphism of MBL2 -221 XX type in exposed control group (general population) as 16%, OR = 2.0, Zα =1.96 for significant level 0.05 and Zβ =0.84 for 80% power. Based on the above assumptions, 186 patients and 210 healthy controls were enrolled.

### Genotyping of MBL2 gene:

Genomic DNA was obtained from whole blood by using commercially available DNA extraction kit (Quick- g DNATM, USA) as per manufacturer's protocol. 100 ng of DNA was used as template for subsequent PCR reactions. Genotyping of MBL2 gene at site codon54: rs1800450 and -221: rs7096206 was done by polymerase chain reaction followed by restriction fragment length polymorphism with specific primer pairs and restriction enzymes. For codon 54: primers used for amplification were; forward 5'GTAGGACAGAGGGCATGCTC3' reverse 5'CAGGCAGTTTCCTCTGGAAGG3’ with a product size of 329 base pair. PCR product was successively amplified at 94°C for 5-minutes, 94°C for 40-seconds, 58°C for 40-seconds, 72°C for 40-seconds and final extension at 72°C for 7 minutes. For promoter site -221: primers used for amplification were; forward 5'ATGCTTACCCAGACAAGCCTGT3' reverse 5'GGTTAATCTCAGTTAATGAACACATATTGGCC3’ with a product size of 608 base pair [13,14]. PCR product was successively amplified at 95°C for 5-minute, 95°C for 30-sec, 58°C for 20-sec, 72°C for 45-sec and final extension at 72°C for 7 minutes. PCR products were further digested by restriction enzyme Ban1and Btg1 (NEB Inc., USA), respectively. The digestion reaction system was kept at 37°C overnight. Finally, the digested PCR products were separated by 2% agarose gel electrophoresis. Gel imaging processing system was used to observe the electrophoresis results of the digested PCR products to determine the genotype.

### Statistical analysis:

Genotype data for control group were analyzed for fitness in the Hardy-Weinberg equilibrium using the online calculator available at http://ihg.gsf.de/cgi-bin/hw/hwa1.pl. Comparison of genotype and allele frequencies in patients and controls was calculated by chi-square test. Confidence interval (CI) and Odd ratio (OR) were also calculated by chi square test. The quantitative variables were analyzed by calculation of mean and standard deviation values, while analyses of categorical variables were done by calculating frequencies and percentages. Continuous groups were compared by Student's t-test while categorical by chi-square (χ^2^) test. P values less than 0.05 (p<0.05) were considered as statistically significant. All the data were analyzed with SPSS software (windows version 17.0; IBM corp., IL, USA).

## Results

A total of 396 demographically-matched subjects were enrolled, of which 186 were FC cases and 210 were controls. The mean age was 37.70±0.81 years (range 18-60 years) for FC patients and 35.98±0.77 years (range 18-60 years) for controls. There were no significant differences in mean age, sex and residence distribution between FC patients and controls (p>0.05), suggesting that matching was adequate (Table 1). The clinical and biochemical parameters were documented only in cases (Table 2).

The frequency of genotype AA, AB and BB at codon54 in FC patients was found to be 107 (57.5%), 61 (32.8%) and 18 (9.7%), while in controls it was 132 (62.9%), 64 (30.5%) and 14 (6.7%), respectively. No significant association was observed between case and control group for variation at codon-54 and susceptibility to FC. Although the rare allele frequency in cases was higher than controls but it failed to attain statistical significance. In addition, allelic frequencies of MBL2 at codon54 showed no significant differences between the two groups (p>0.05).

The frequency of YY, YX and XX genotypes of MBL2-221 gene in patients with FC was found to be 95 (51.1%), 82 (44.1%) and 9 (4.8%), while in control subjects it was 93 (44.3%), 84 (40.0%) and 33 (15.7%), respectively. The mutant genotype showed significant odds in cases than controls (Odds ratio=0.27, 95% Confidence interval=0.12-0.59, p<0.001) for protection against FC (Table 3).

## Discussion

The population of endemic region is exposed to mosquitoes but only some acquire infection. The population in our study was from an area that is endemic to filariasis. We planned the present study to find out the genetic factors that might influence the risk for an individual to develop FC. In spite of the fact that FC is a common public health problem affecting several regions of the world, it has been neglected by researchers. Unlike other diseases, genetic aspects of this disease have not been looked into seriously by researchers. MBL, a serum protein synthesized by liver that plays a key role in innate immunity. MBL binds to surface of oligosaccharides of bacteria, viruses and parasites and induces the compliment system via interaction with MBL-associated serine protease, and facilitates phagocytosis and opsonization of pathogens [7]. Studies have shown that deficiency of MBL2 causes defective phagocytosis and opsonization, resulting in several diseases including LF [8,9]. Various SNPs in MBL2 gene have been reported, but only some have functional relevance. Variations at amino acid coding region may result in deformed proteins which degrade leading to its lower levels, while variations at promoter regions may affect binding of transcription factor and possibly reduce transcript levels that ultimately translate to lesser protein expression. The levels of MBL are affected by promoter polymorphism genotype but the promoter variants do not alter function of protein. Host genetic factors can determine differences in susceptibility or resistance to infections, as well as in the clinical pattern of diseases. In our study we noticed a possible link between base pair -221(XX) genotype (p=<0.001) polymorphism with FC infection. In our observations -221 polymorphism showed significantly higher frequency of variant genotypes in controls (15.7%) as compared to FC patients (4.8%) (p = <0.001). Similarly, Choi et al. [9] also reported that this variant genotype was more frequent among controls (16%) in comparison to cases (4.0%). In addition some other reports on the high frequency of MBL mutant allele in general population suggested that MBL2 deficiency may be associated with certain life-threatening diseases [15,16]. Our results showed no significant association between structural MBL2 codon 54 variants and FC infection, suggesting that these variants possibly did not have any association with outcome of disease in our study population.

We noticed that FC group has lower frequency of AA genotypes of MBL2 gene codon 54 (57.5% versus 62.9%), and higher frequency of AB and BB genotype (32.8% versus 30.5%) and (9.7% versus 6.7%) when compared with control group. While rare allele frequency in FC group was higher than controls but it failed to attain statistical significance. In control group, we found that prevalence of AB and BB genotypes of MBL2 codon 54 polymorphism was 30.5% and 6.7%, respectively, and distribution of YX and XX genotypes of MBL2 promoter (-221) was 40% and 15.7%, respectively. In study from south India, the frequency of MBL2 polymorphisms (-221) in healthy controls were 33% and 16%, respectively, which is fairly similar to our observation [9].

The associations of these polymorphisms with other diseases have also been explored. A study on systemic lupus erythromatous patients reported that the frequency in healthy controls of MBL2 polymorphisms (codon 54 and -221) YX and XX genotypes were 30% and 17%, and AB and BB genotypes were 14% and 5%, respectively [17]. In another study on malaria patients, Jha et al, reported that the genotype frequency of YX and XX in healthy controls were 31.2% and 6.4% and AB and BB were 10.2% and 0.6%, respectively [18]. There are some limitations in our study. Out of the different manifestations of LF we chose only FC. This was done because urologists (study was conducted in Department of Urology), of the various manifestations, primarily manage chyluria. Other common manifestations like hydrocele and elephantiasis of penis and scrotum are also sometimes encountered by urologist. However, at our center these are managed primarily by general surgeons and plastic surgeons. That is the reason why we limited our study population to chyluria only. There are limitations in the serological tests used for the diagnosis of filarial etiology. Some of these limitations are because of the long natural history of filariasis and also because chyluria is a chronic manifestation of LF. Several other polymorphisms have been reported in MBL2 gene However, only two (codon 54 and promoter -221) were included in the present investigation to correlate it with predisposition to FC infection.

Existing literature suggests a minor or no roles of promoter variants in diseases; only promoter variants -221 and structural variant codon 54 are clinically important [19]. The prevalence of codon 52 and 57 mutants is rare in North Indian population. In a study, the reported gene frequencies of codon 54, 52 and 57 were 15%, 5% and 2%, respectively, in HIV-1 infected patients [20]. Another study carried out on 213 subjects of systemic lupus erythromatous in the province of Odisha in India reported only three patients to be heterozygous for codon 52 and one for codon 57 [17]. We did not analyze the impact of other genes. More gene polymorphism site and large sample size will be analyzed in the future. Serum analysis of MBL levels was not performed. Thus, we were unable to assess whether serum MBL levels altered during infection. Our study is a preliminary study; there is need for more studies to confirm our findings. However, the strength of our study is that we have explored a condition on the literature is scanty. Our study will help in understanding the patho physiological basis of this important but neglected disease that afflicts millions of people.

## Conclusion

We report the significant association of MBL2-221 promoter polymorphism with the susceptibility to FC infection. However, this is not true for the polymorphism at codon 54. These observations are interesting requiring further confirmation using a larger study with more patients.

## Figures and Tables

**Table 1:** Demographic characteristics of study subjects:

Basic characteristics	Cases (n=186)(%)	Controls (n=210)(%)	p value
Age (yrs):			
Mean ± SD	37.70 ± 0.81	35.98 ± 0.77	0.124
Sex:			
Female	56 (30.1)	53 (25.2)	0.279
Male	130 (69.9)	157(74.8)	
Location:			
Urban	60 (32.3)	83 (39.5)	0.133
Rural	126 (67.7)	127(60.5)	

**Table 2:** Clinical and biochemical parameters of Filarial chyluria patients

Clinical characteristics	FC (n=186) (%)
Grade of chyluria:	
I	65 (34.9)
II	84 (45.2)
III	37 (19.9)
Duration of history of chyluria (months)	52.04±5.16 (1-396)*
Number of previous episodes (number)	2.29±0.11 (0-7)*
Duration of current episode (days)	9.61±0.48 (2-20)*
Urine Triglycerides (g/dl)	239.87±15.68 (10-1193.5)*
Urine Cholesterol (g/dl)	23.36±2.65 (0.5-369)*
Haemoglobin (g/dl)	11.63±1.4 (7.4-13)*

**Table 3:** Distribution of genotypic and allele frequency of gene polymorphism in filarial chyluria patients and controls

Genotype/ Allele	Cases	Controls	OR	P-Value
	(n=186) (%)	(n=210) (%)	(95% CI)	
MBL2 codon 54:				
Genotype				
Wild (AA)	107 (57.5)	132 (62.9)	Ref	
Hetero (AB)	61 (32.8)	64 (30.5)	1.18 (0.76-1.81)	0.464
Mutant (BB)	18 (9.7)	14 (6.7)	1.59 (0.75 -3.34)	0.224
AB+BB	79 (42.5)	78 (37.1)	1.25 (0.84-1.87)	0.279
MBL2 codon 54:				
Allele				
A	275 (73.9)	328 (78.1)	Ref	
B	97 (26.1)	92 (21.9)	1.26 (0.90 -1.74)	0.169
MBL2-221:				
Genotype				
Wild (YY)	95 (51.1)	93 (44.3)	Ref	
Hetero (YX)	82 (44.1)	84 (40.0)	0.96 (0.63-1.45)	0.831
Mutant (XX)	9(4.8)	33(15.7)	0.27(0.12-0.59)	<0.001
YX+XX	91 (48.9)	117 (55.7)	0.76 (0.51-1.13)	0.177
MBL2-221:				
Allele				
Y	272 (73.1)	270 (64.3)	Ref	
X	100 (26.9)	150 (35.7)	0.66 (0.49-0.90)	0.008
